# Supplementary material for: Linked dimensions of psychopathology and connectivity in functional brain networks
Source: Nat Commun. 2018 Aug 1;9:3003. doi: 10.1038/s41467-018-05317-y (PMC6070480; doi:10.1038/s41467-018-05317-y)
Supplement: Supplementary file 1 — Supplementary Information [file 41467_2018_5317_MOESM1_ESM.pdf]

Supplementary Information | Linked dimensions of psychopathology and connectivity in functional brain networks

1. **Supplementary Figures and Legends 1–12**;
2. **Supplementary Tables 1-2**;

Supplementary Information

Supplementary Figures

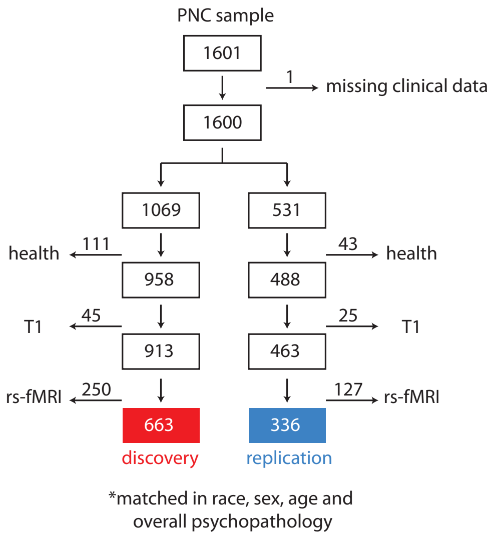

Supplementary Figure 1 | Sample Construction. The cross-sectional sample of the Philadelphia Neurodevelopmental Cohort (PNC) has 1601 participants in total. Excluding the one missing clinical data, 1600 participants were randomly stratified into a discovery (n=1069) and a replication sample (n=531). Applying quality exclusion criteria for health, structural imaging, and functional imaging (details in Online Methods), 663 and 336 subjects were included in the final discovery and replication samples, respectively.

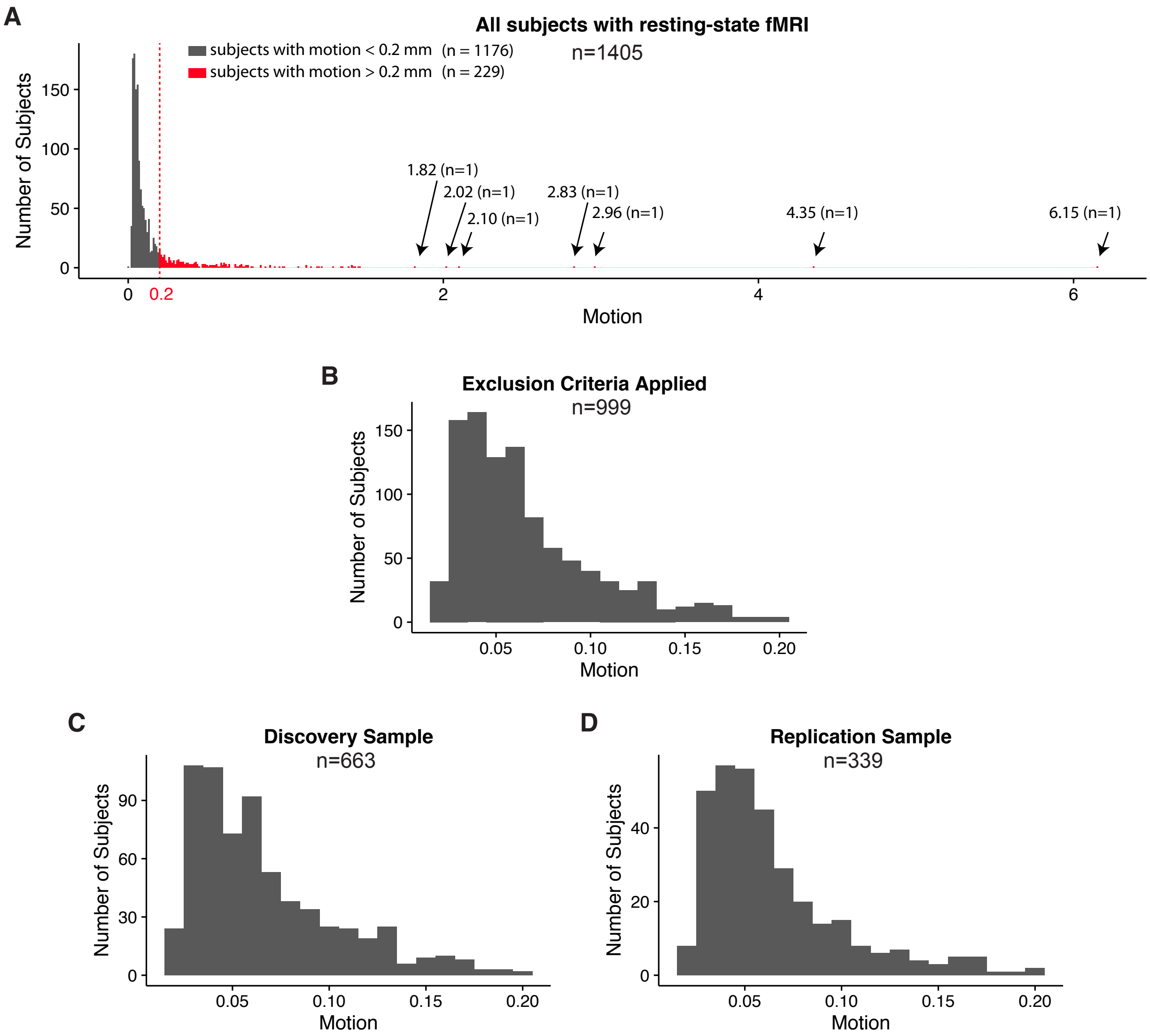

Supplementary Figure 2 | **In-scanner motion of subjects . (a)** 1405 out of 1601 participants of PNC had acquired resting-state fMRI. The histogram shows the distribution of mean framewise displacement using the Jenkinson calculation. The exclusion criteria of motion for the final sample is 0.2mm or greater, which is colored in red (n=229). **(b)** After applying all exclusion criteria, including health, structural and functional imaging quality exclusion criteria, 999 subjects were included in the final sample. The histogram shows the head motion distribution of the final sample, which consists of a discovery sample **(c)**, and a replication sample **(d)**.

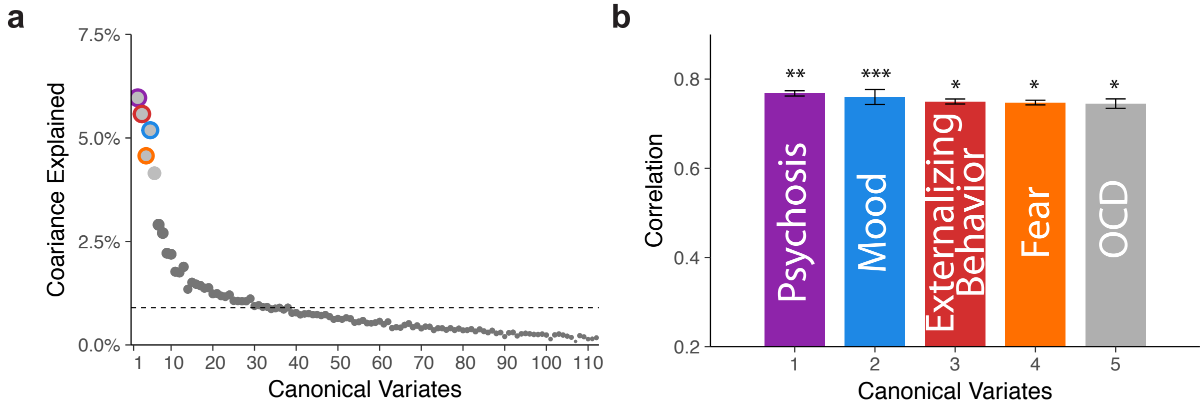

Supplementary Figure 3 | Pre-processed data without global signal regression (GSR). (a) We preprocessed the functional data with 12 parameter + *aCompCor*,which is the one of the best performing preprocessing procedures to correct for motion without GSR.[1–4] Subsequently, we followed the same procedures as in the main analysis. The first five canonical variates were selected for further analysis based on covariance explained. Dashed line marks the average covariance explained. **(b)** The original four dimensions -— psychosis, mood, fear, and externalizing behavior — and a fifth dimension (corresponding to OCD-spectrum symptoms) were significant by permutation testing. Corresponding variates are circled in panel (a). Error bars denote standard error. *** $P_{\mathrm{FDR}} < 0.001$, ** $P_{\mathrm{FDR}} < 0.01$, * $P_{\mathrm{FDR}} < 0.05$.

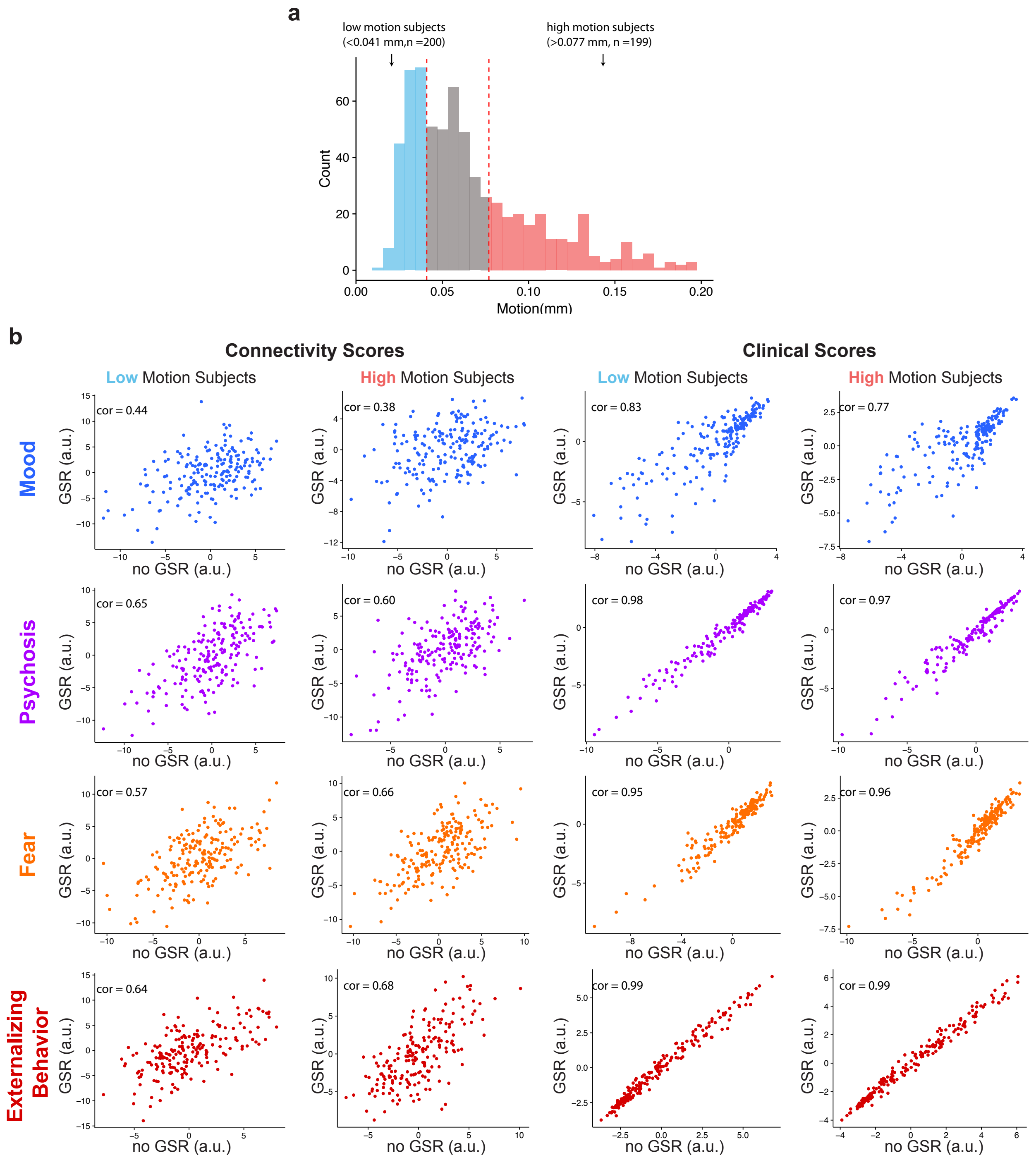

Supplementary Figure 4 | Comparison of GSR effects in low and high motion subjects. (a) Histogram of subject in-scanner motion in the discovery cohort (n=663), of which those with the lowest motion (< 0.041 mm, n = 200) and those with the highest motion (> 0.077 mm, n =199) were selected for the comparison of their CCA dimensional scores processed with and without GSR. **(b)** We calculated the correlation coefficient between the CCA dimensional scores (i.e. connectivity and clinical scores) processed without GSR (x axis) and those processed with GSR (y axis) in each motion group for each of the four canonical dimensions. All correlation coefficients were highly significant ($P < 2.2 \times 10^{-16}$).

A

median absolute deviation (MAD)

$$= \text{median}(\ |\mathbf{X}_i - \text{median}(\mathbf{X})|\ )$$

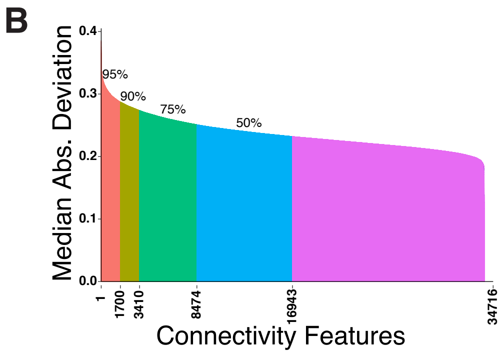

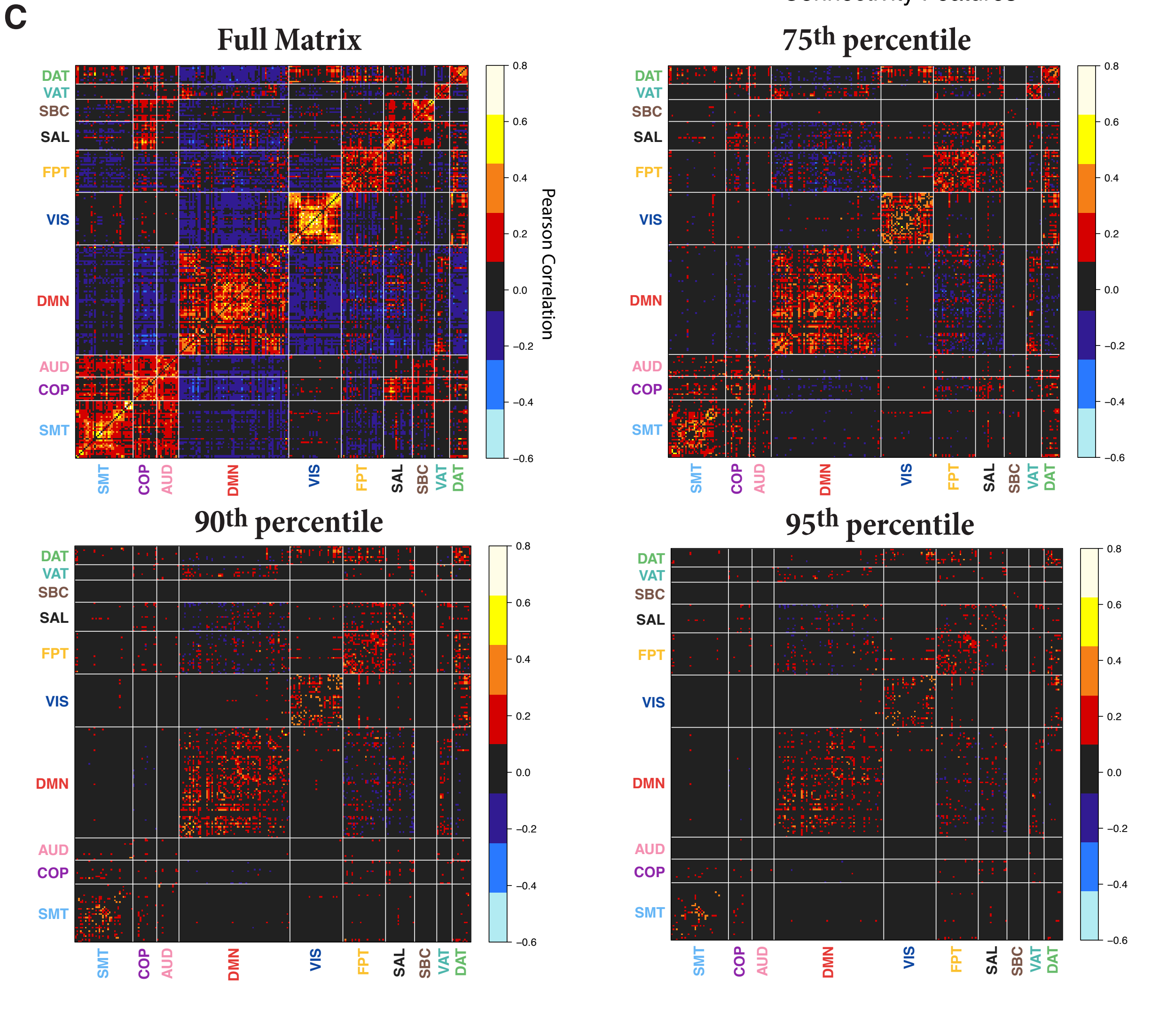

Supplementary Figure 5 | Connectivity feature selection using median absolute deviation (MAD). Since sCCA seeks to capture sources of variation common to both datasets, we selected top 10% or 3410 connectivity features that were variable across the discovery sample. **(a)** The variance was calculated using the median absolute deviation (MAD). It is defined as the median of the difference between each element and the median in a vector. **(b)** MAD of each edge strength in decreasing order. The 95th, 90th, and 75th percentile are labeled, where the 90th corresponds to 3410 edges. **(c)** Average connectivity matrix across all participants of edges with MAD at 100th, 95th, 90th, and 75th percentile levels.

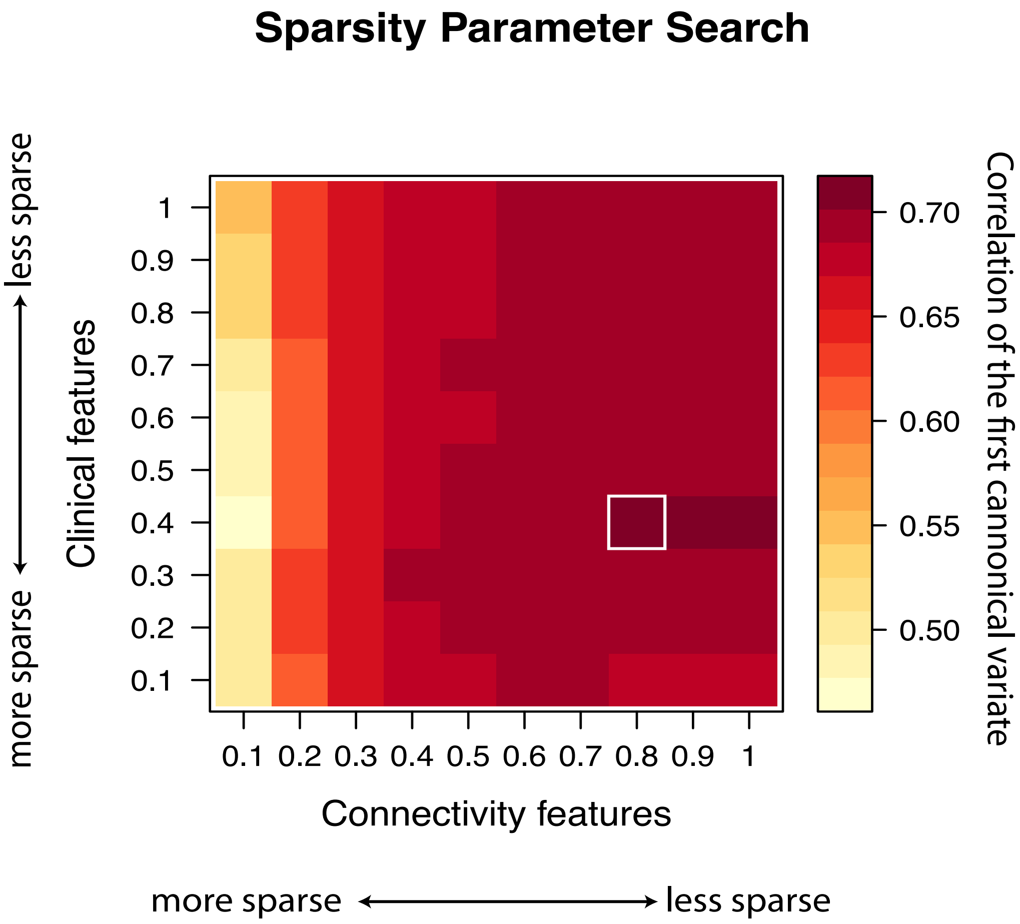

Supplementary Figure 6 | Grid search for regularization parameters. We tuned the L^1 regularization parameters for the connectivity and the clinical features in sCCA. The range of sparsity parameters is constrained to be between 0 and 1 in the PMA package,[5] where 0 indicates the smallest number of features (i.e. highest level of sparsity) and 1 indicates the largest number of features (i.e. lowest level of sparsity). We conducted a grid search in increment of 0.1 to determine the combination of parameters that would yield the highest canonical correlation of the first variate across 10 randomly resampled datasets, each consisting of two-thirds of the discovery dataset.

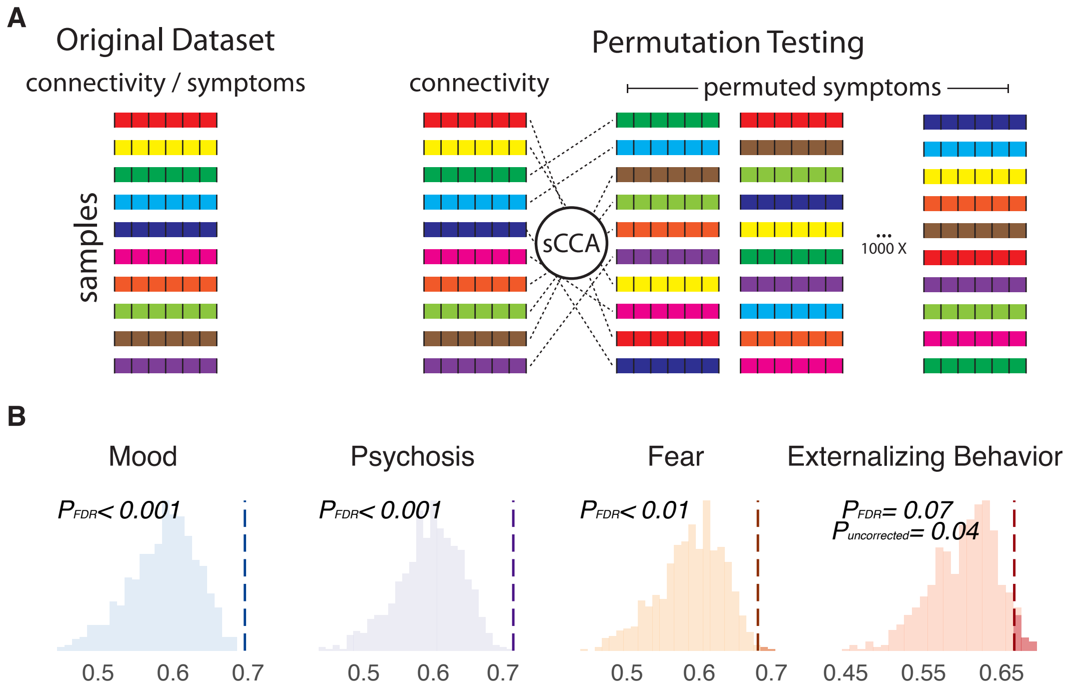

Supplementary Figure 7 | Permutation testing to assess significance of linked dimensions. (a) Schematic of permutation procedure. Connectivity data was held constant, while the rows of the clinical matrix were randomly shuffled, so as to break the linkage of participants' connectivity features and their symptom features. As permutation could induce arbitrary axis rotation, which changes the order of canonical variates, or axis reflection, which causes a sign change for the weights, we matched the canonical variates resulting from permuted data matrices to the ones derived from the original data matrix by comparing the clinical loadings (**v**).[6] **(b)** Null distributions of correlations generated by the permuted data. Dashed line represents the correlation from the original dataset.

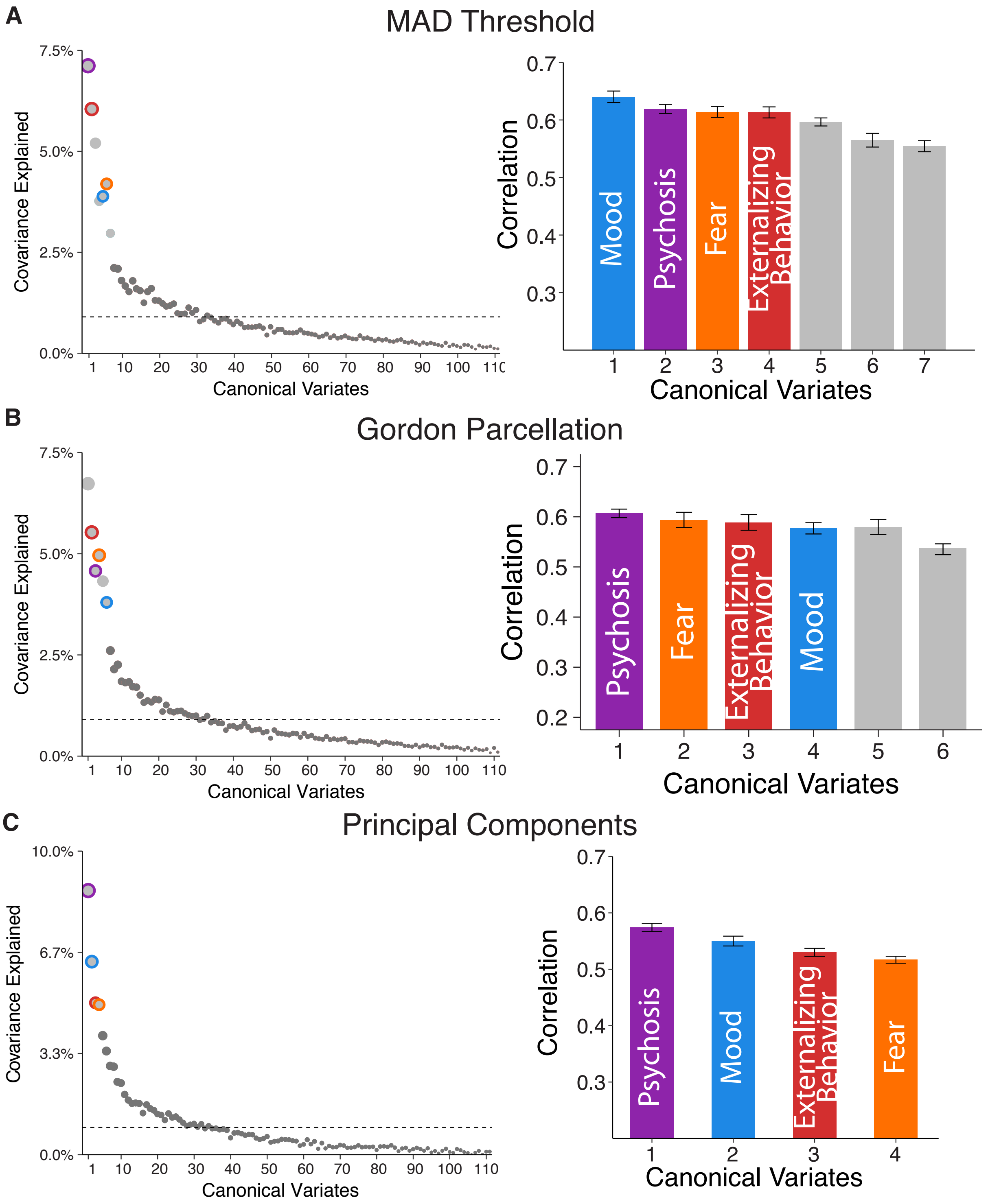

Supplementary Figure 8 | **Patterns of canonical variates were robust to methodological choices.** We found four canonical variates based on covariance explained and correlation across methodological choices, including **(a)** the number of features entered into the analysis (edges with top 5% variance based on MAD), **(b)** an alternative parcellation (Gordon et al.[7]), and **(c)** using alternative techniques of dimensionality reduction (the first 111 principal components). Dashed line marks the average covariance explained. Corresponding variates on the right panels are circled in the left. Error bars denote standard error.

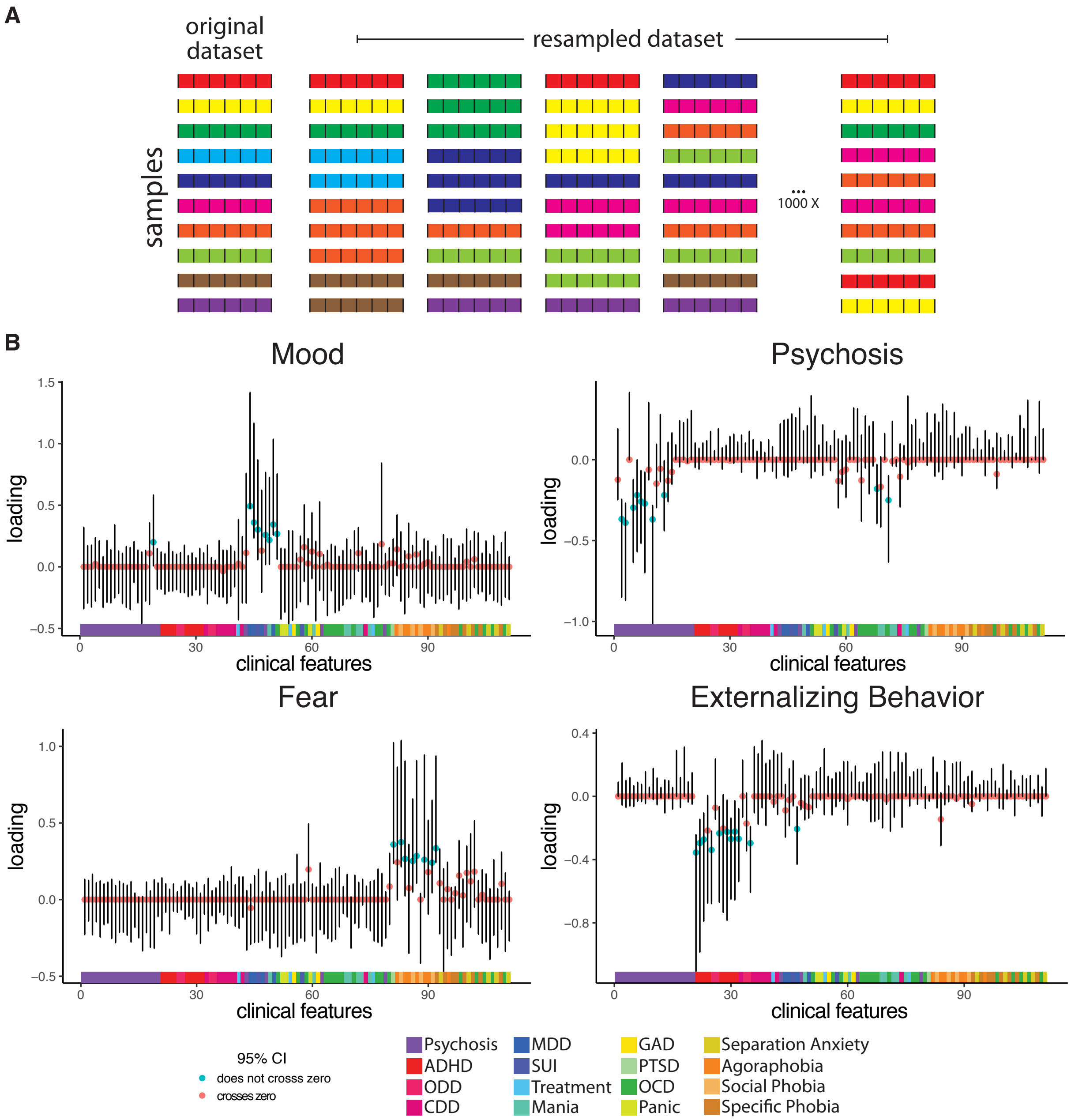

Supplementary Figure 9 | Resampling procedure to identify stable features contributing to each linked dimension. **(a)** Schematic of the resampling procedure. In each sample, two-thirds of the discovery dataset was first randomly selected. The sample size was completed to be the same as the original by replacing with those already selected. **(b)** Resampling distribution for clinical features in each linked dimension. Each bar represents the 95% confidence interval. DSM categories to which each symptom item belongs are shown.

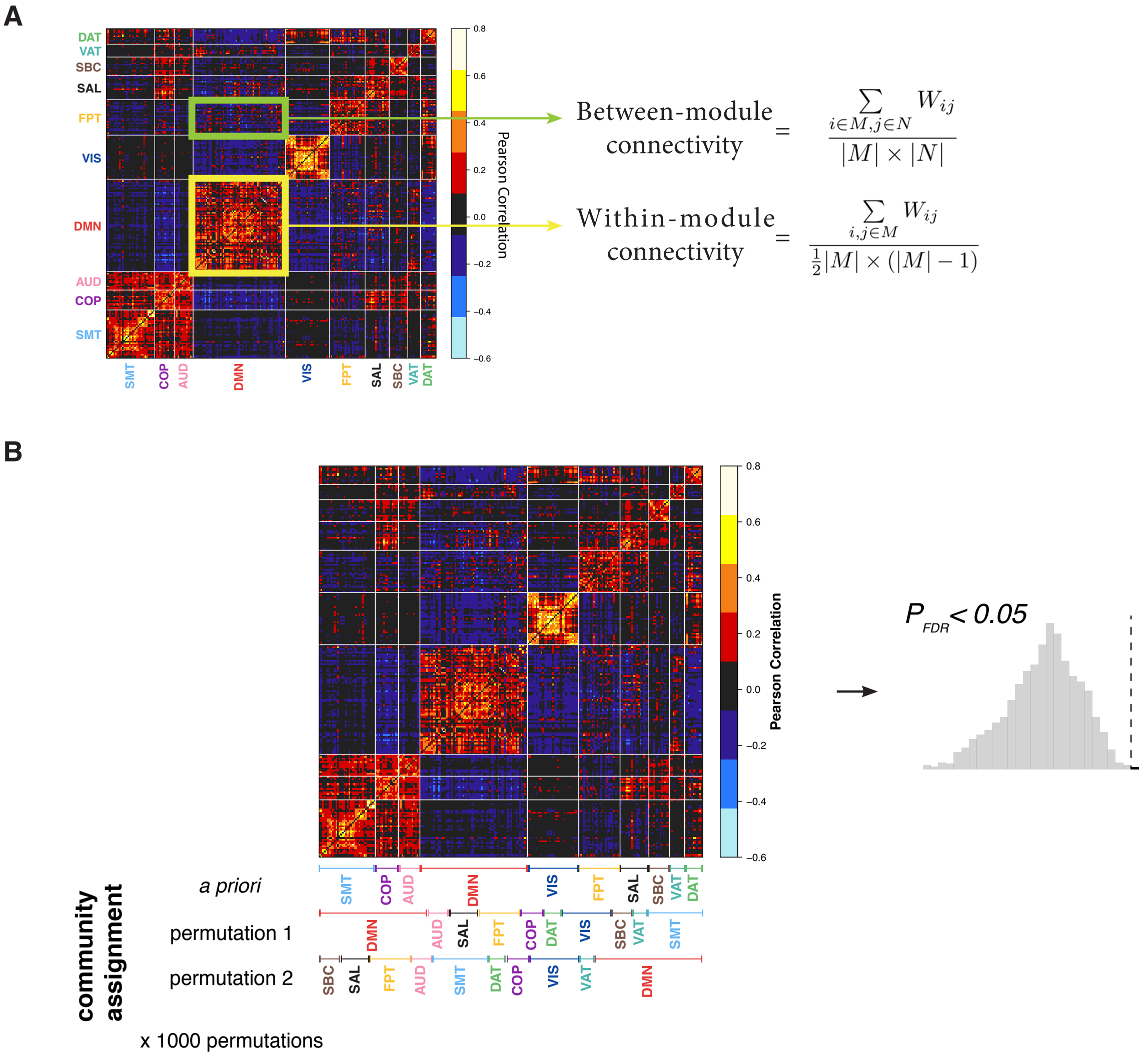

Supplementary Figure 10 | **Network module analysis.** **(a)** Summarizing loadings on a between- and within-network basis using *a priori* community assignment from the parcellation of Power et al.[8] **(b)** Schematic for generating null model for modular analysis. Community membership was randomly assigned to each node while controlling for community size. Mean between- and within-module loadings were then calculated based on these permuted modules, which we used to assess the statistical significance by comparing the orginal values against the null distribution.

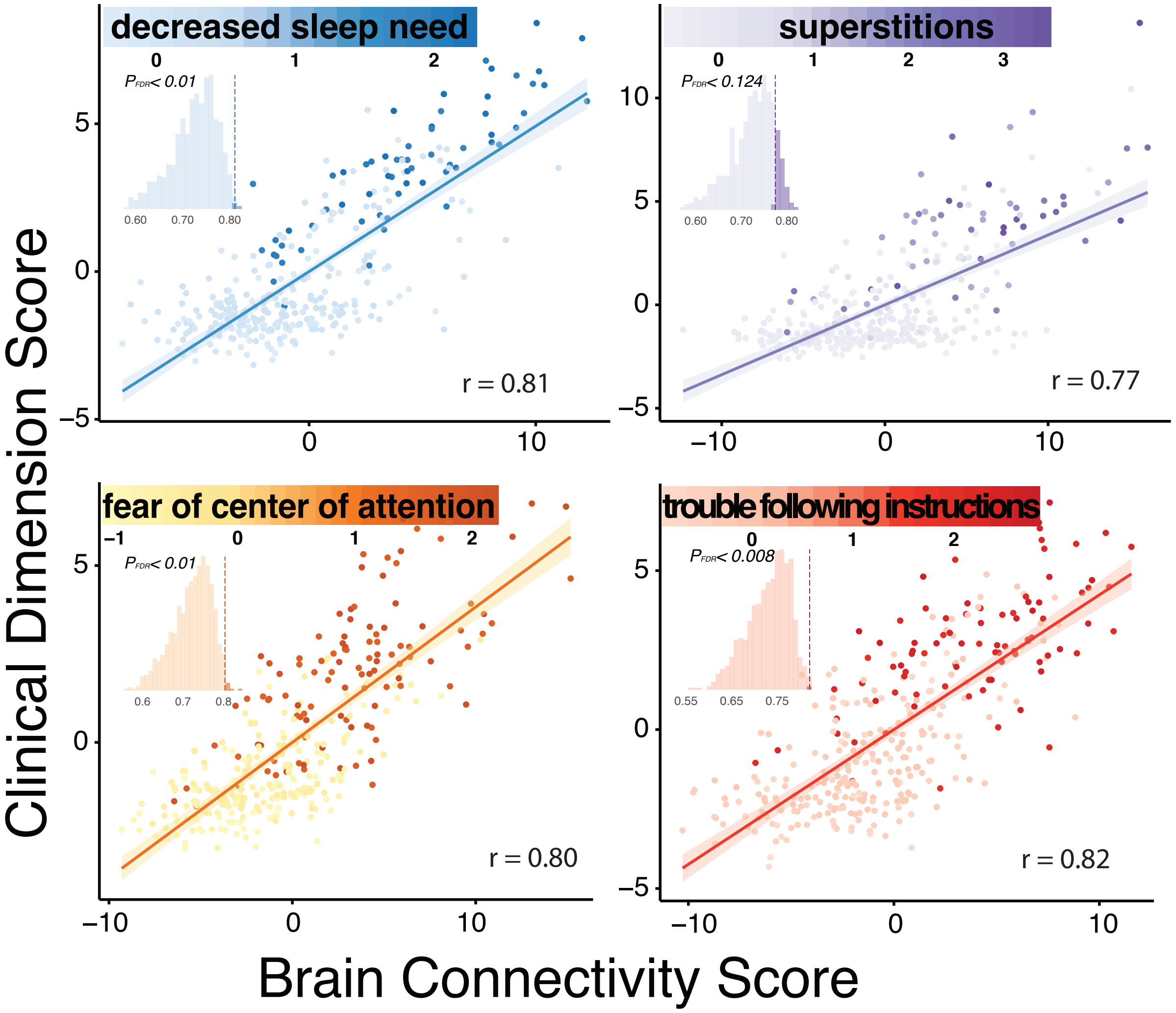

Supplementary Figure 11 | Canonical variates in the replication sample accord with findings in the discovery sample. Scatter plots of brain and clinical scores (linear combinations of functional connectivity and psychiatric symptoms, respectively) demonstrate the correlated multivariate patterns of connectomic and clinical features. Colored dots in each panel indicate the severity of a representative clinical symptom that contributed the most to this canonical variate. Each insert displays the null distribution of sCCA correlation by permutation testing. Dashed line marks the actual correlation.

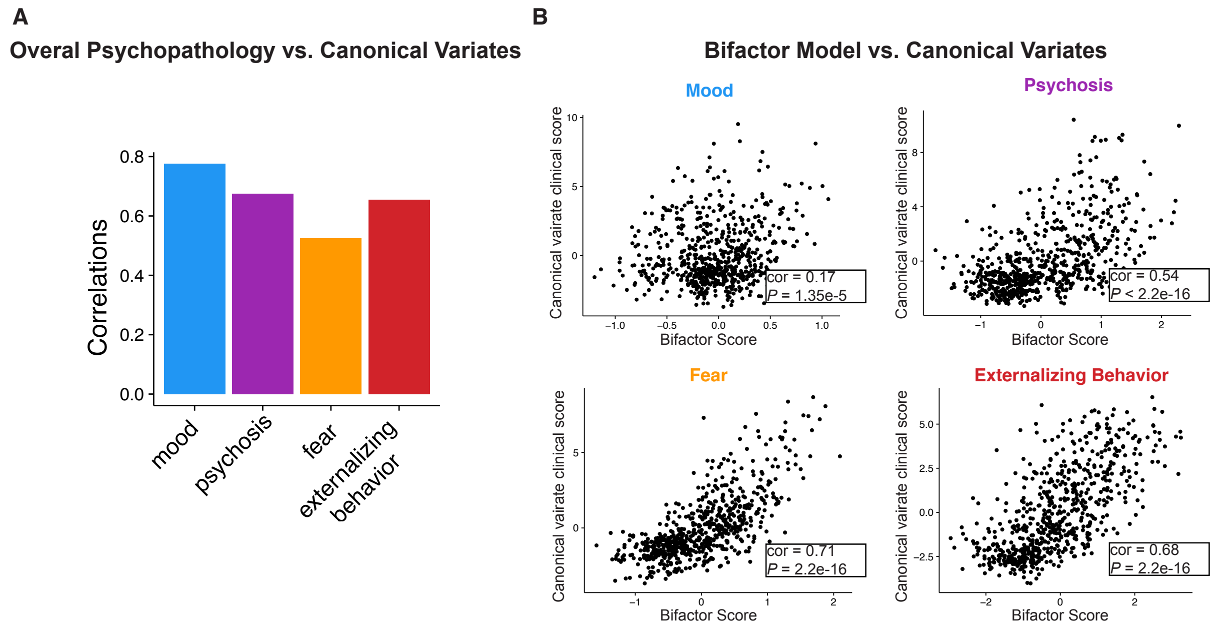

Supplementary Figure 12 | Correlations between canonical variates and previous factor analysis model. To understand how similar connectivity-guided dimensions of psychopathology are to those derived from pure clinical items reported before, we examined the correlation between the canonical variate clinical scores and **(a)** overall psychopathology score, and **(b)** dimensional bifactor models scores, both initially reported in Shanmugan et al.[9]

Supplementary Tables

Supplementary Table 1 | Clinical Psychopathology Levels in the PNC

**Psychopathology Categories**	All sample (n=1601)	Total analyzed sample (n=999)	Discovery (n=663)	Replication (n=336)
Mania	1.2%	1.3%	1.1%	1.8%
Depression	13.2%	15.3%	16.3%	13.4%
Bulimia	0.4%	0.3%	0.5%	0.0%
Anorexia	1.0%	1.4%	1.8%	1.2%
Generalized Anxiety Disorder	1.7%	1.6%	1.8%	1.2%
Separation Anxiety Disorder	4.7%	3.9%	4.1%	3.6%
Social Phobia	23.1%	24.8%	25.2%	24.1%
Panic Disorder	1.0%	0.8%	1.1%	0.3%
Agoraphobia	5.7%	5.8%	6.5%	4.5%
Obsessive-Compulsive Disorder	2.8%	2.7%	2.6%	3.0%
Post-Traumatic Stress Disorder	11.7%	12.4%	12.4%	12.5%
Psychosis	6.7%	6.6%	7.5%	4.8%
Attention-Deficit Disorder	17.3%	15.3%	15.2%	15.5%
Conduct Disorder	8.9%	8.6%	7.8%	10.1%

Supplementary Table 2 | Correlations of loadings between covariate-regressed and non-regressed features

	Connectivity	Symptoms
Mood	0.73	0.54
Psychosis	0.95	0.88
Fear	0.70	0.35
Externalizing behavior	0.98	0.97

Loadings of both connectivity and clinical features across dimensions were highly correlated between input data that had age and sex regressed out of and those that had not. All correlations were statistically significant ($P_{\text{FDR}} < 0.001$).

References

1. Behzadi, Y., Restom, K., Liau, J. & Liu, T. T. A component based noise correction method (CompCor) for BOLD and perfusion based fMRI. *NeuroImage* **37**, 90–101 (2007).

2. Muschelli, J. *et al.* Reduction of motion-related artifacts in resting state fMRI using aCompCor. *NeuroImage* **96**, 22–35 (2014).

3. Ciric, R. *et al.* Benchmarking of participant-level confound regression strategies for the control of motion artifact in studies of functional connectivity. *NeuroImage* **154**, 174–187 (2017).

4. Parkes, L., Fulcher, B. D., Yucel, M. & Fornito, A. An evaluation of the efficacy, reliability, and sensitivity of motion correction strategies for resting-state functional MRI. *bioRxiv* 156380 (2017).

5. Witten, D. M., Tibshirani, R. & Hastie, T. A penalized matrix decomposition, with applications to sparse principal components and canonical correlation analysis. *Biostatistics* **10**, 515–534 (2009).

6. Mišić, B. *et al.* Network-Level Structure-Function Relationships in Human Neocortex. *Cerebral Cortex* **26**, 3285–3296 (2016).

7. Gordon, E. M. *et al.* Generation and Evaluation of a Cortical Area Parcellation from Resting-State Correlations. *Cerebral Cortex* **26**, 288–303 (2016).

8. Power, J. D. *et al.* Functional network organization of the human brain. *Neuron* **72**, 665–78 (2011).

9. Shanmugan, S. *et al.* Common and Dissociable Mechanisms of Executive System Dysfunction Across Psychiatric Disorders in Youth. *The American journal of psychiatry* **173**, 517–526 (2016).
