# Supplementary material for: Linked dimensions of psychopathology and connectivity in functional brain networks
Source: Nat Commun. 2018 Aug 1;9:3003. doi: 10.1038/s41467-018-05317-y (PMC6070480; doi:10.1038/s41467-018-05317-y)
Supplement: Supplementary file 3 — Description of Additional Supplementary Files [file 41467_2018_5317_MOESM3_ESM.pdf]

Description of Additional Supplementary Files:

Supplementary Data 1: A long table that contains the details of the 111 clinical items used in the study, including the DSM category, item labels and specific questions from the GOASSESS semi-structured interview.
